# Longitudinal Study of Circulating Biomarkers in Patients with Resectable Pancreatic Ductal Adenocarcinoma

**DOI:** 10.3390/bios12040206

**Published:** 2022-03-30

**Authors:** Pablo J. Dopico, Minh-Chau N. Le, Benjamin Burgess, Zhijie Yang, Yu Zhao, Youxiang Wang, Thomas J. George, Z. Hugh Fan

**Affiliations:** 1 Interdisciplinary Microsystems Group, Department of Mechanical and Aerospace Engineering, University of Florida, Gainesville, FL 32611, USA; ufpablo@ufl.edu (P.J.D.); minhchaunle@ufl.edu (M.-C.N.L.); 2 UF Health Cancer Center, University of Florida, 2033 Mowry Rd., Gainesville, FL 32610, USA; benjamin.burgess@ufl.edu; 3 Atila Biosystems, 740 Sierra Vista Ave., Unit E, Mountain View, CA 94043, USA; zhijie.yang@atilabiosystems.com (Z.Y.); shoemakerzhao@gmail.com (Y.Z.); ywang@atilabiosystems.com (Y.W.); 4 Department of Medicine, University of Florida, 1600 SW Archer Rd., Gainesville, FL 32610, USA; 5 J. Crayton Pruitt Family Department of Biomedical Engineering, University of Florida, Gainesville, FL 32611, USA

**Keywords:** circulating tumor cells, circulating cell-free DNA, circulating tumor DNA, pancreatic cancer, microfluidics

## Abstract

While patients with resectable pancreatic ductal adenocarcinoma (PDAC) show improved survival compared to their non-resectable counterparts, survival remains low owing to occult metastatic disease and treatment resistance. Liquid biopsy based on circulating tumor cells (CTCs) and cell-free DNA (cfDNA) has been shown to predict recurrence and treatment resistance in various types of cancers, but their utility has not been fully demonstrated in resectable PDAC. We have simultaneously tracked three circulating biomarkers, including CTCs, cfDNA, and circulating tumor DNA (ctDNA), over a period of cancer treatment using a microfluidic device and droplet digital PCR (ddPCR). The microfluidic device is based on the combination of filtration and immunoaffinity mechanisms. We have measured CTCs, cfDNA, and ctDNA in a cohort of seven resectable PDAC patients undergoing neoadjuvant therapy followed by surgery, and each patient was followed up to 10 time points over a period of 4 months. CTCs were detectable in all patients (100%) at some point during treatment but were detectable in only three out of six patients (50%) prior to the start of treatment. Median cfDNA concentrations remained comparable to negative controls throughout treatment. ddPCR was able to find KRAS mutations in six of seven patients (86%); however, these mutations were present in only two of seven patients (29%) prior to treatment. Overall, the majority of circulating biomarkers (81% for CTCs and 91% for cfDNA/ctDNA) were detected after the start of neoadjuvant therapy but before surgery. This study suggests that a longitudinal study of circulating biomarkers throughout treatment provides more useful information than those single time-point tests for resectable PDAC patients.

## 1. Introduction

Despite improvements to the mortality of several cancer types over the past few decades, pancreatic ductal adenocarcinoma (PDAC) continues to have a poor 5-year survival of 4–7% [1]. Patients for whom PDAC is detected early enough, called resectable PDAC, can be treated with a combination of surgery and chemotherapy resulting in improved survival outcomes [2,3]. However, even among these patients, survival rates remain low (18–23.8%) partially owing to occult metastatic disease and treatment resistance [4].

The diagnosis and prognosis of bloodborne biomarkers such as circulating tumor cells (CTCs), cell-free DNA (cfDNA), or the tumorigenic subset of cfDNA, known as circulating tumor DNA (ctDNA), are often referred to as liquid biopsy [5]. The presence and concentration of these circulating biomarkers have been shown to correspond to cancer recurrence and chemotherapy resistance for various carcinomas including PDAC [6,7].

CTCs are tumor cells that break off from a primary tumor and intravasate into the blood stream. Of liquid biopsy biomarkers, CTCs represent ongoing metastasis because of their ability to form new tumors in a distant location [8]. In PDAC patients treated with neoadjuvant (pre-operative) therapy, patients with elevated CTCs after treatment were more likely to suffer recurrence [7]. Similarly, resectable PDAC patients who underwent surgical resection were more likely to have liver metastasis after a 3-year follow-up if CTCs were present in the portal system after surgery [6].

Detecting CTCs is challenging due to their rarity in the blood (often ≤ 10 cells/mL) compared to red blood cells (~10^9^ cells/mL) and white blood cells (~10^7^ cells/mL) [6,9,10,11,12,13,14]. Currently, the only FDA approved CTC detection technology, CellSearch (Menarini Silicon Biosystems, Castel Maggiore BO, Italy), is limited by low sensitivity and variability [12,15]. We previously reported a lateral filter array microfluidic (LFAM) device, which integrated immunoaffinity with filtration for CTC isolation [11]. The LFAM device has a higher CTC capture efficiency than devices based on immunoaffinity only or filtration only.

cfDNA is any free-floating DNA in plasma regardless of origin [16]. Non-tumorigenic cells can release DNA into plasma during apoptosis, injury, or disease. Despite the lack of specificity, cfDNA is known to correspond to tumor burden. While typically lower compared to non-resectable PDAC patients, cfDNA is still elevated in resectable patients when compared to healthy controls [17,18,19]. In a group of PDAC patients that included resectable patients, increased cfDNA concentrations were shown to correspond to metastasis, vascular encasement, and lower survival [17]. A conflicting study, however, found no relationship between cfDNA concentration and survival [19].

ctDNA is a subset of cfDNA that is specifically of tumorigenic origin. In the case of PDAC, tumorigenic DNA can be identified through the presence of a KRAS mutation, which are present in >90% of PDAC [19]. Similar to increased cfDNA concentrations, the presence of ctDNA has been linked to statistically shorter overall survival (OS) in some instances [19] and no statistical difference in others [17]. One likely contributor to the inconsistency in results between ctDNA presence and survival relates to the amount of blood collected as a pre-analytic variable. For example, the relatively low detection rate of KRAS mutations in plasma increases with the decreasing blood volume drawn. In typical studies, only 19–30% of resectable patients expressed a KRAS mutation at blood draw prior to treatment [17,18,19]. While another study managed to find a KRAS mutation in 49% of resectable patients at baseline; this required 40 mL blood draws, far above the amount drawn in other studies [20].

While liquid biopsy biomarkers have been shown to have some clinical utility in resectable PDAC patients, further work is needed to establish their usefulness in treatment monitoring. Currently, most liquid biopsy studies combine all types of patients with PDAC (resectable and non-resectable patients) [17,18,19,21]. Since circulating biomarkers are likely to be lower in resectable PDAC patients than in non-resectable PDAC patients, relationships between clinical outcomes and the presence of circulating biomarkers in these studies may not apply to resectable PDAC. Additionally, only a few studies exist tracking either CTCs [7] or cfDNA [20] over multiple time points, with most studies only detecting these biomarkers over one or two time points [6,17,18,19]. When including studies over multiple time points, no studies assess the change of circulating biomarkers over the course of neoadjuvant therapy prior to surgery. Studies in other cancer types have demonstrated that CTCs and cfDNA concentrations, as well as the presence of ctDNA mutations, can change throughout therapy, presumably as a dynamic response to treatment.

In this prospective study, we present our preliminary findings on the detection of three liquid biopsy biomarkers in resectable PDAC patients undergoing neoadjuvant therapy as well as post-surgery. We utilize a single 10-mL blood sample to detect both cfDNA and CTC concentrations as well as the presence of ctDNA mutations through a combination of LFAM devices [10,11], quantitative polymerase chain reaction (qPCR), and droplet digital PCR (ddPCR).

## 2. Methods

**Patient Recruitment and sample collections.** In compliance with the University of Florida Institutional Review Board (IRB201703333), patients with potentially resectable PDAC were recruited as part of a larger study investigating neoadjuvant multi-agent chemotherapy prior to surgical resection. Patients underwent 8 cycles of treatment (~2 weeks/cycle, a total of 4-month treatment) with the combination of 5-fluorouracil, leucovorin, oxaliplatin and irinotecan. After 8 cycles, patients underwent surgery for tumor removal. Up to 10 mL of whole blood was drawn during each visit prior to treatment as well as after surgical resection, for a maximum of 10 blood draws, per patient. The first blood draw was immediately prior to treatment initiation and is hereafter referred to as the baseline or cycle 1 sample. Blood was also drawn prior to surgery, referred to as pre-operative blood draw or cycle 9, and again after surgery, referred to as post-operative (final) blood draw or cycle 10.

TNM (tumor, node, metastasis) classification of malignant tumors staging defined by the American Joint Committee on Cancer (AJCC) was done at baseline (clinical staging) and operatively (pathologic staging). Additionally, patients were given a treatment score [1,2,3] based on their response to therapy. Where a score of 3 indicated no evident tumor regression and a score of 1 corresponded to only single or small groups of cancer cells remaining after treatment [22,23]. This scoring system is described by the College of American Pathologists (CAP) and is unlikely to be effected by observer bias compared to similar scoring systems [22]. Common terminology criteria for adverse events (CTCAE) was used to score toxicity for patients over the duration of treatment [24].

Tumor specimens collected at the time of surgery underwent CLIA-approved comprehensive next generation sequencing consistent with clinical standards of care (Tempus xT assay; Tempus, Inc.; Chicago, IL, USA). De-identified profiling reports were used to confirm the presence or absence of a mutation that is theoretically present in the blood. In addition to PDAC patients, a number of “cancer free” controls had blood drawn. These subjects were PDAC survivors currently in complete remission without evidence of disease after a minimum of 3 years; hence, they are referred to as “PDAC survivors” in subsequent discussion.

**Reagents.** Dulbecco’s phosphate buffered saline (DPBS) with 0.49 mM magnesium chloride and 0.9 mM calcium chloride, DPBS containing 1% bovine serum albumin (BSA), and ethanol were purchased from Fisher Scientific (Hampton, NH, USA). A Sylgard-184 silicone elastomer was bought from Dow Chemical (Midland, MI, USA). Epithelial cell adhesion molecule (EpCAM) antibody was bought from eBioscience (San Diego, CA, USA). A KRAS Wild-Type and a KRAS G12D DNA reference standard were purchased from Horizon Discovery (Waterbeach, UK). CaPan-1 cells (HTB-79, pancreatic adenocarcinoma) were purchased from American Type Culture Collection (ATCC, Manassas, VA, USA), while MDA-MB-231 cells (HTB-26, breast adenocarcinoma) were donated from Dr. Dietmar Siemann (Department of Radiation Oncology, University of Florida). CaPan-1 and MDA-MB-231 were used as positive controls for G12V [25] and G13D [26] KRAS mutations, respectively.

All cells were cultured in T25 flasks (Corning Inc., Corning, NY, USA) in a 5% CO_2_ environment at 37 °C. CaPan-1 cells were cultured using high glucose IMEM supplemented with 20% heat inactivated fetal bovine serum (FBS, Corning Inc.). MDA-MB-231 cells were cultured in high glucose DMEM with 4.0 mM L-glutamate media (DMEM, GE Healthcare) supplemented with 10% FBS and 100 units/mL of Penicillin-Streptomycin. Tumor cells isolated from peripheral blood using anti-EpCAM were verified using the FDA-approved CTC criterium, that is, EpCAM^+^DAPI^+^CK^+^CD45^-^ after staining with 4′6-diamidino-2-phenylindole (DAPI, Fisher Scientific) and immunostaining with both anti-cytokeratin-fluorescein isothiocyanate (anti-CK-FITC, BD Bioscience, San Jose, CA, USA) and anti-CD45-phycoerythrin (anti-CD45-PE, BD Biosciences).

**Device Fabrication and Preparation.** Silicon masters of LFAM devices were fabricated as previously described [10,11]. Individual substrates of polydimethylsiloxane (PDMS) containing microchannels and other features were fabricated from a silicon master with a Sylgard-184 silicone elastomer. Each PDMS substrate was separated from the silicon master after curing, washed with ethanol (EtOH) followed by DI water, and then dried by an air gun. Both the substrate and a microscope slide were then treated with UV ozone for 5 min, before the substrate was gently pressed onto the surface of the microscope slide to create a finished LFAM device.

The LFAM device was then coated with anti-EpCAM for CTC capture (Figure S1). First, EtOH was introduced into the device at 2 µL/s to wet the channels. A DPBS volume of 250 µL was then introduced at 2 µL/s to wash out EtOH prior to the introduction of avidin. 100 µL of 2 mg/mL avidin was incubated within the LFAM device for 20 min at room temperature. Afterwards, excess avidin was washed out with 250 µL of DPBS at 1 µL/s. 100 µL of 20 µg/mL biotinylated anti-EpCAM was then incubated within the device for 20 min at room temperature. Finally, 250 µL of 1% BSA was incubated in the device for at least one hour to reduce non-specific cell binding.

As shown in Figure S1, the LFAM device utilizes a combination of filtration and immunoaffinity to capture CTCs from the blood of patients [11]. The device consists of 4 serpentine channels whose walls are made of a series of lateral filters. The lateral filters reduce possible clogging compared to conventional vertical filters in which the flow is in the same direction of filtration. The lateral filtration in LFAM also allows the filter sizes to decrease from 10 µm near the inlet to 6 µm near the outlet (Figure S1), enabling selective capture of different sizes of CTCs. As tumor cells attempt to squeeze through lateral filters, they interact with antibodies immobilized on the filter surfaces and become captured. White blood cells and red blood cells do not interact with antibodies and can therefore squeeze through the filters due to their elasticity and/or smaller sizes.

**Blood preparation and CTC capture.** Up to 10 mL of patient blood from an EDTA-tube was transferred to a 50-mL centrifuge tube previously incubated with 1% BSA, and then centrifuged at 2000× *g* for 10 min, separating the blood into cellular and plasma components. The plasma component was carefully pipetted into a new 15-mL centrifuge tube, which was centrifuged once again at 2000× *g* for 10 min, resulting in 4–5 mL of plasma free of cellular components. 2 mL of plasma was aliquoted and immediately frozen to −80 °C prior to being shipped overnight for ddPCR mutation detection, while another 2 mL of plasma was used immediately for qPCR to measure the cfDNA concentration and determine KRAS mutations.

The cellular portion of blood was resuspended with a volume of DPBS equal to the original blood volume plus the volume of plasma removed, resulting in a 2× dilution of the original blood sample. Up to 4 mL of this sample was passed through an LFAM device coated with anti-EpCAM at 1 µL/s, followed by a 450-µL DPBS wash at 2 µL/s. Afterwards, cells were fixed with 4% paraformaldehyde (PFA) for 10 min, washed, and then permeabilized with 0.2% Triton-X for 10 min. Captured cells were stained for DAPI, anti-CK, and anti-CD45 in order to distinguish CTCs from non-specifically captured white blood cells (WBCs). Cells were considered CTCs if they were roughly circular, DAPI^+^, CK^+^, and CD45^−^ (Figure S2), consistent with the definitions adopted by the U.S. Food and Drug Administration (FDA).

**qPCR cfDNA measurement.** For qPCR, cfDNA was extracted from plasma using a QIAamp DNA Blood Mini Kit (Qiagen; Hilden, Germany) according to the manufacturer’s instructions, except with volume differences noted here. 2 mL of plasma was used for cfDNA extraction along with 2 mL of buffer AL and 200 µL of proteinase K (New England Biolabs; Ipswich, MA, USA). DNA was eluted in a 120-µL volume.

To estimate cfDNA concentrations in patients, a standard curve was constructed from a known concentration of wild-type (WT) KRAS reference standard (Horizon Discovery) serially diluted at a 1:5 ratio (Figure S3). In addition to WT KRAS, detection of G12D, G12V, and G13D KRAS mutations were carried out. These mutations were selected as they represent the most common KRAS mutations in PDAC patients [27]. To establish reference curves for each KRAS mutation, 10^6^ positive control cells expressing these KRAS mutations were used (Figure S4).

qPCR was carried out in triplicates of 25 µL volumes. Each 25-µL sample consisted of 5 µL of eluted DNA, 12.5 µL of SYBR Green Master Mix (Applied Biosystems; Foster City, CA, USA), and 7.5 µL of either WT or mutation specific primers. For mutation detection, the primer mix consisted of 2.5 µL of forward primer, reverse primer, and WT blocker at 3 nmol/mL, as described in the literature [28]. For WT KRAS only, forward and reverse WT primers were used and 2.5 µL of nuclease free water was added to bring up the primer volume to 7.5 µL. qPCR was carried out on a Quant Studio 3 Real Time PCR System (Applied Biosystems, CA, USA). The denaturation and annealing temperature for each cycle of qPCR was set at 95 °C and 60 °C. After 40 cycles, the reaction temperature was decreased to 60 °C and then slowly increased to 90 °C at a rate of 0.15 °C/s to establish a melting curve.

The cycle of quantification (C_q_) value of wild-type KRAS was compared to the standard curve to determine the concentration of cfDNA for each patient at each treatment cycle. Patients were considered positive for a KRAS mutation if the melting temperature of the reaction matched those of positive controls [28] (Figure S5) and the C_q_ of the reaction was ≤38 cycles for G12D or G13D and ≤39 for G12V. This cutoff is based on the Minimum Information for Publication of Quantitative Real-Time PCR Experiments (MIQE) guidelines [29] because it was the lowest C_q_ for which we could still detect >95% of samples at the correct melting temperature.

**ddPCR.** Droplet digital PCR (ddPCR) was carried out at Atila Biosystems (Mountain View, CA, USA). ddPCR and qPCR methods were developed and carried out independently, without knowing each other’s results before experiments. cfDNA was extracted from 2 mL of human plasma using a QIAamp MinElute ccfDNA Midi Kit (Qiagen; Hilden, Germany) according to the manufacturer’s instructions with an elution volume of 40 µL.

ddPCR was carried out using the KRAS Multiplex Mutation Screening Kit (Atila Biosystems; Mountain View, CA, USA) according to the manufacturer’s instructions. 1–2 ng of cfDNA and 2 µL of primers were added to 10 µL of ddPCR^TM^ Supermix (BioRad; Hercules, CA, USA), forming a 20 µL ddPCR reaction. Samples were prepared and run as duplicates. For each run of ddPCR, a no-template control was included.

ddPCR droplets were generated using a QX200^TM^ Droplet Generator (BioRad) according to the manufacturer’s instructions. ddPCR was then run for 60 cycles with a denaturation temperature of 95 °C for 15 s and an annealing temperature of 60 °C for 50 s. 60 cycles were performed to ensure that all samples reached the plateau phase of ddPCR. After 60 cycles, the reaction temperature was raised to 98 °C for 10 min before being cooled to room temperature and read using a QX200^TM^ Droplet Reader (BioRad). Taqman fluorescence probes labeled with either 6-carboxyflourescein (FAM) or hexachloroflourescein (HEX) were used to identify WT or MT DNA. In the presence of WT DNA, both FAM and HEX probes cleave and fluoresce due to exonuclease activity, while in the presence of MT DNA, only HEX signal is present (Figure S22). This allowed for the separation of distinct clusters representing negative samples, WT, and MT DNA (Figure S23). No cross reactivity between WT and MT was observed when the assay was carried out using WT DNA. Positive signal threshold was based on amplification amplitude once samples reached the plateau phase. To calculate the percent mutant allele frequency (%MAF), the concentration of MT KRAS was divided by the concentration of WT KRAS.

## 3. Results

**Patient Staging.** Seven patients with newly diagnosed, previously untreated and potentially resectable PDAC participated in the study described here. At diagnosis, all cancer patients were T_2_, N_0_, M_0_ by AJCC TNM staging classification. An equal number of PDAC survivors participated and served as negative controls. One patient (P003) was removed from the study due to treatment complications. The treatment was continued at a later date, after which the patient underwent surgery and tumor sequencing approximately seven months after the last blood draw. All others completed therapy and proceeded to surgery. Four patients (P001, P004, P006, and P007) additionally had radiation therapy following completion of chemotherapy but prior to surgery. Following neoadjuvant chemotherapy and at the time of surgery, no patient had developed metastasis (M_0_), three patients had lymph node invasion (N_1_), two patients showed tumor shrinkage (T_1_) and two had slight tumor growth (T_3_). For the CAP treatment score, two patients had significant improvement (score 1) and only one patient had no improvement (score 3). There was no observable relationship between TNM staging at the surgical stage and CAP treatment scores (Table 1 and Table S1).

**CTC enumeration.** All laboratory personnel for CTC enumeration, qPCR, and ddPCR analysis were blinded to patient status and outcome. In total, 57 blood samples were received for analysis, 46 of which (or 81%) had CTCs enumerated and 52 of which (or 91%) had cfDNA and ctDNA analysis performed. In some instances, blood was not drawn due to patient complications, insufficient blood was collected, or COVID-19 preventing sample analysis. One patient (P001) did not have a baseline blood sample available, and two patients (P003 and P007) did not have a post-operative blood sample available.

**Figure 1:**
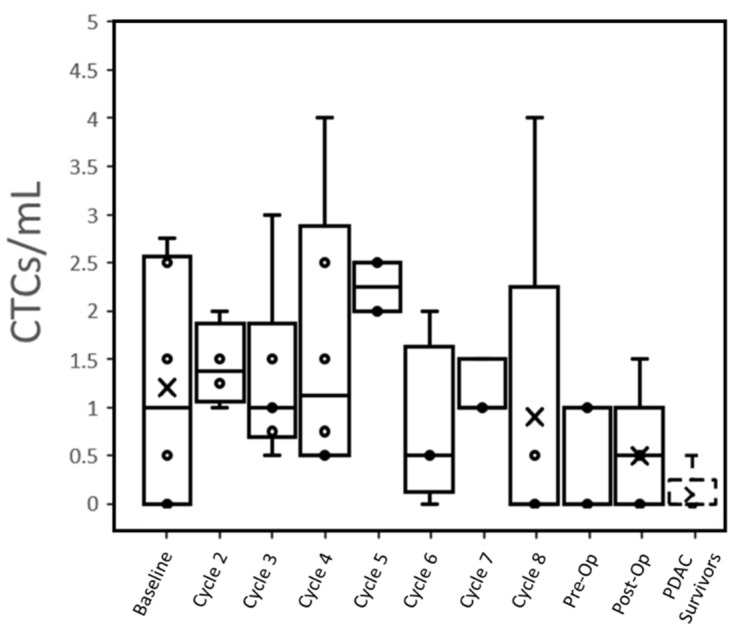
Trend in the CTC number per mL of patient blood samples over the treatment cycles, pre-operation (pre-OP) and post-operation (post-OP). Each treatment cycle is 2 weeks. Patient data was pooled and plotted as a box plot for each cycle. The whiskers represent the minimum and maximum; the box bottom, the bar inside the box, and the box top represent lower quartile, median, and upper quartile; the cross represents the average; and each circle represents one or multiple datapoints depending on if CTC numbers are overlapping (see the detail in Figures S7–S13). PDAC: pancreatic ductal adenocarcinoma.

At baseline, 66% (4 of 6) patients had CTCs that were detected in the peripheral blood (1.2 ± 1.2 CTC/mL, Table 2). This reduced to 60% (3 of 5) of patients post-operatively (0.5 ± 0.6 CTC/mL, Figure 1). Only one PDAC survivor out of five controls (note: only qPCR was performed in two other controls) had a potentially detectable CTC (≤0.5 CTC/mL), while all active PDAC patients expressed >0.5 CTC/mL at one or more time points (Figures S7–S13). When pooled together, no substantial difference in CTC counts over time was seen between patients with different N-staging or treatment score (Figure S6).

Individual trends suggested that radiation therapy may reduce the number of CTCs expressed by patients (Figures S7–S13). The CTCs/mL of all four patients who underwent radiation therapy dropped to 0 in the subsequent blood draw. Even individually, no relationship could be seen between CTC concentrations immediately, pre-operatively and with subsequent TNM surgical staging. Both patients with a CAP treatment score of 1 showed a steady decline in CTCs over time, but this trend was observed across all patients regardless of score.

**cfDNA concentration**. According to qPCR results, only one out of six patients at baseline had a cfDNA concentration greater than the maximum cfDNA concentration observed in PDAC survivor control samples. The maximum cfDNA concentration of PDAC survivors was preferred to the average cfDNA concentration in order to identify when cancer patients had a cfDNA concentration outside of the range of PDAC survivors. Furthermore, there is no statistical difference based on t-test in cfDNA concentration measure by qPCR for PDAC survivors and cancer patients (p: 0.77, Table 2).

**Figure 2:**
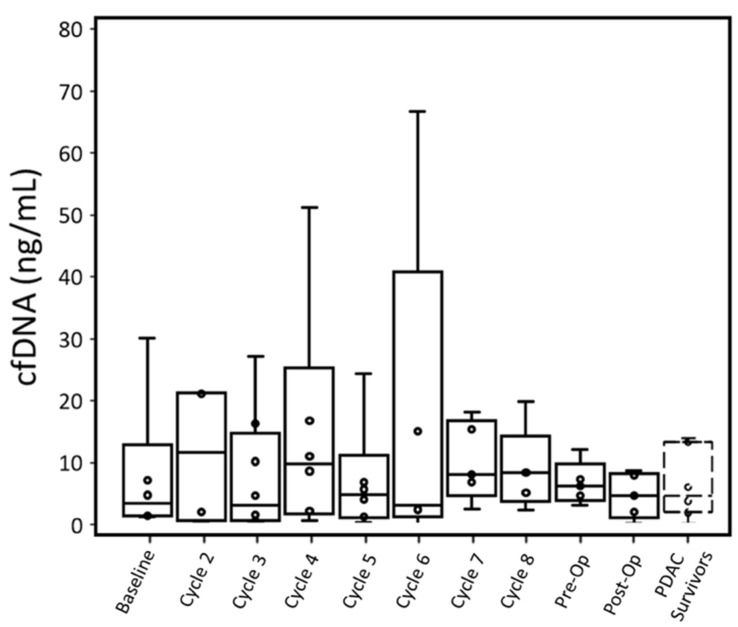
Trend of cfDNA concentration of all patients measured by qPCR over the treatment cycles, pre-operation (pre-OP) and post-operation (post-OP). PDAC survivor samples are included for comparison. The whiskers represent the minimum and maximum; the box bottom, the bar inside the box, and the box top represent lower quartile, median, and upper quartile; the cross represents the average; and each circle represents one data point.

**Figure 3:**
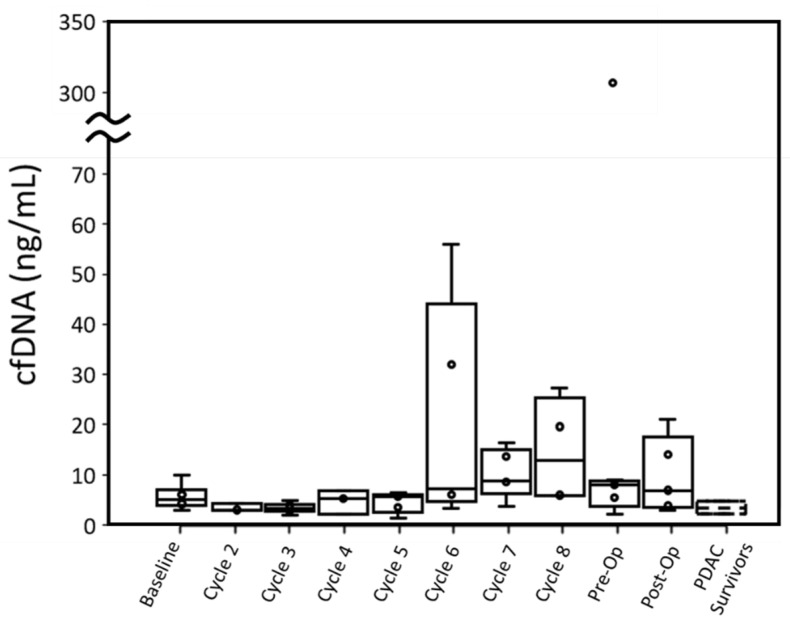
Trend of cfDNA concentration of all patients measured by ddPCR over the treatment cycle, pre-operation (pre-OP) and post-operation (post-OP). PDAC survivor samples are included for comparison. The whiskers represent the minimum and maximum; the box bottom, the bar inside the box, and the box top represent lower quartile, median, and upper quartile; the cross represents the average; and each circle represents one data point.

Four out of six patients had cfDNA greater than PDAC survivors at baseline when measured with ddPCR. This difference in cfDNA was statistically significant (p: 0.027). While P001 did not have a baseline blood draw, their cfDNA concentration at cycle 2 was also higher than the cfDNA concentration of PDAC survivors. Overall, throughout treatment, active cancer patients retained a median cfDNA concentration within the range of PDAC survivors, though a few patients displayed high cfDNA concentrations at specific cycles (Figure 2 and Figure 3).

cfDNA concentrations for individual patients were compared between qPCR and ddPCR (Figures S14–S20). With both PDAC survivor controls and cancer patient samples, qPCR tended to overestimate the concentration of cfDNA compared to ddPCR. For PDAC survivors, qPCR had a larger range of cfDNA concentrations (1.96–13.98 ng/mL) than ddPCR (1.5–4.7 ng/mL). Similarly, for PDAC patients, at each time point, qPCR had a larger range of observed cfDNA with the exception of the preoperative time point, which was due to a sample not analyzed with qPCR assay.

Cancer patients’ cfDNA concentrations measured by either assay were similar to PDAC survivors’ throughout most time points, although most patients had one or more time points with substantially elevated cfDNA. For ddPCR, elevated cfDNA typically occurred past cycle 5 (Figure 3). Unlike CTCs, no relationship between radiation therapy and change in cfDNA concentration was observed. High concentrations of cfDNA could not be adequately explained by cytotoxic effects of treatment as high CTCEA events (Table S1) did not always correspond to high cfDNA.

Upward or downward trends in cfDNA did not correlate with T-staging or CAP treatment scores. While patients with lower CAP scores (better treatment response) did express lower cfDNA concentrations throughout treatment, there was no relationship between change (from baseline to pre-op time points) in cfDNA and CAP scores.

In terms of cfDNA concentration measured by qPCR and ddPCR, P004 (12.13 ng/mL and 13.99 ng/mL, respectively) and P007 (10.16 ng/mL and 5.94 ng/mL, respectively) had the lowest peak values among all patients excluding P003. P005 expressed the highest cfDNA peak in ddPCR (307.26 ng/mL) and was unmeasured by qPCR at this time point due to insufficient plasma. This patient had the worst CAP treatment score and gained lymph node metastasis (N_1_) but did not have overall tumor growth.

**ctDNA.** We have studied tissue and plasma samples and compared their KRAS mutation status between tissue sequencing and ddPCR (Table 3). Six out of seven patients expressed a KRAS mutation in tumor tissue. At baseline, two were detected in ddPCR, suggesting that baseline mutation concordance between ddPCR and tumor tissue was low. Additionally, in one patient (P004), a ddPCR mutation was detected whereas no mutation was present in tumor tissue.

For ddPCR assay, two patients (P002 and P004) had a KRAS mutation detected in plasma at baseline blood draw. Three additional patients (P001, P006, and P007) demonstrated a KRAS mutation while undergoing treatment and no patient had a detectable KRAS mutation in either the pre- or post-operative samples. Of these patients, P001, P002, P006, and P007 all had a detectable KRAS G12D mutation in tumor tissue while P004 did not have any mutation present. Two patients (P003 and P005) had no mutation detected by ddPCR at any time point despite the presence of tumor mutations. In all cases, the no template control failed to amplify, suggesting that these results were not due to non-specific amplification.

## 4. Discussion

Our results demonstrate that we can successfully detect CTCs in early stage (e.g., resectable) PDAC patients, including during their treatment with neoadjuvant chemotherapy. These CTC results are substantially higher than those using CellSearch while being similar to those reported by alternative methods [6,19,30,31]. Bissolati et al. only found CTCs in 4/20 resectable patients using CellSearch when drawing from the systemic forearm [6]. In a much larger study including 99 pancreatic cancer patients, CTCs were detected in only 8% of Stage II patients with CellSearch [30]. These reports are lower than what we observed in our patients (7/7) using microfluidic devices based on both immunoaffinity and filtration. However, our observation is in line with publications using more sensitive CTC detection methods. For example, NanoVelcro, an immunoaffinity based CTC capture method, was able to detect CTCs in 23/31 of stage II patients and 4/9 stage I patients [31,32].

In our study, CTCs were detected less frequently and at lower concentrations than our similar study of metastatic, unresectable PDAC patients [21]. The range of CTCs was lower for resectable PDAC patients both at baseline (0–2.75 CTCs/mL versus 4.5–8.25 CTCs/mL in metastatic patients) and at the last time point of chemotherapy (0–1 CTCs/mL versus 1–4 CTCs/mL). While CTC retrieval of metastatic PDAC utilized an immunoaffinity microfluidic device, the lower concentration of CTCs in resectable PDAC patients cannot be attributed to device differences, as we previously demonstrated that the LFAM device has higher capture efficiency than the immunoaffinity device utilized in the previous study [11].

While it has been demonstrated that CTCs can be useful in monitoring treatment response in other cancers [21,33,34,35], the low concentration of CTCs we observed in our early stage PDAC compared to metastatic PDAC [21] will likely make it difficult to correlate CTC changes with treatment response. In our cohort of patients, CTC counts decreased to near the range as PDAC survivors by the time of the post-operative blood draw (Figure 1), suggesting that treatments were successful in eradicating the source of the CTCs.

When compared to clinical outcomes, we cannot correlate CTC numbers with lymph node invasion or a pathology regression score (Figure S6) due to high variability between CTC counts and the insufficient sample size in our study that prevent us from performing adequate statistical analysis. CTC detection and concentration have been inconsistently demonstrated to be predictive of lymph node invasion in some epithelial cancers [13,36]. In a study of PDAC patients of different stages and metastatic presence, the presence of CTCs did not correspond to lymph node invasion [37]. Our results are consistent with this finding for resectable PDAC patients.

Individual patient trends are provided in Figures S7–S13. For treatment score, patients with a good response (Score 1) had a decrease in CTCs from the baseline to the end of treatment, although a decrease in CTCs over the course of treatment was ubiquitous among patients. Individually, no relationship could be seen between CTC concentrations at immediate pre-operation and with subsequent TNM surgical staging.

Our results suggest that radiotherapy decreases the number of CTCs detected in resectable PDAC patients. The CTCs/mL of all four patients who underwent radiation therapy dropped to 0 in the subsequent blood draw. Decreasing CTC concentration in response to radiotherapy has been demonstrated in other cancer types [35,38], which is consistent with our observations.

While we observed changes in CTC counts over the course of treatment, most of these changes were relatively small. This makes it difficult to draw any conclusions between clinical measures of treatment success and CTC concentration over time. A previous study utilizing CellSearch demonstrated that most PDAC patients do not have large changes in CTCs as a response to treatment [37]. Since our patient population involves non-metastatic PDAC, and CTCs are primarily a biomarker of ongoing metastasis, it is possible that even the inclusion of additional samples would not result in a difference in CTC concentration by clinical outcomes.

Both our qPCR and ddPCR assays reported lower concentrations of cfDNA than those cfDNA values in the literature [17,18]. Singh et al. used an identical method for cfDNA extraction as our qPCR, but reported a much larger range (15–240 ng/mL) and median (72 ng/mL) of cfDNA [17] even when comparing at equivalent time points. Likewise, a study by Pietrasz et al. reported a much higher amount of cfDNA (mean 52.5 ± 79.5 ng/mL) in resectable PDAC patients than our ddPCR assay, despite using equivalent extraction methods [18]. The difference between these two studies and our effort is likely due to both studies in the literature using UV absorbance to measure cfDNA concentration instead of more specific qPCR or ddPCR. Both studies in the literature, however, demonstrate that cfDNA is elevated in non-metastatic or resectable PDAC patients relative to healthy controls, which is consistent with our results.

Although our qPCR standard curve retains a high R squared value (0.98), the discrepancy in measured cfDNA concentration between qPCR and ddPCR is better explained by the differences of two methods. ddPCR is an absolute quantification method, theoretically returning the exact number of wild-type and mutated DNA present in the sample, while qPCR is an estimate based on a standard curve. Therefore, discrepancies in the measured cfDNA concentration between the two assays can be explained by the higher errors of the qPCR assay. At low concentrations of DNA, as is the case in our study, qPCR suffers from high errors due to stochastic effects. As a result, we regard the ddPCR measurements as more conclusive than the qPCR assay.

While our results demonstrate higher cfDNA concentrations in PDAC patients over our controls (PDAC survivors), it is possible that PDAC survivors have elevated liquid biomarkers compared to healthy individuals with no history of cancer. Therefore, the inclusion of healthy individuals with no history of cancer would further support our results. Nevertheless, our previous study [21] using heathy subjects show zero CTCs among 84.2% controls (16/19), which is comparable to this study (zero CTCs among 80% controls or 4/5).

Most studies of cfDNA in resectable PDAC patients focus on cfDNA concentration at a single or few time points such as baseline draw or after surgical resection. We observed several time points where cfDNA concentration was elevated in patients relative to PDAC survivors in both qPCR and ddPCR between the start of treatment and prior to surgical resection that cannot be explained by cytotoxic effects of treatment. Previous literature has shown that cfDNA may become temporarily elevated as a result of chemotherapy or radiotherapy [16,39]. If temporary elevations in cfDNA concentration (as shown in Figure 3) is indicative of tumor necrosis due to successful treatment, then this information is lost by limiting PDAC patient sampling to baseline, pre-operative, and post-operative blood draws. Further study is needed to determine if short-term elevations of cfDNA are indicative of successful treatment in resectable PDAC patients.

When compared with ddPCR, our qPCR assay seemed to lack the sensitivity to detect KRAS mutations in patient samples. This lack of sensitivity is not likely due to errors in the qPCR methodology as we observed 100% concordance between our qPCR assay and our ddPCR assay in a cohort of CRC patients (Figure S21). This is likely due to the increase in cfDNA and ctDNA concentration in the late-stage CRC patients, resulting in higher odds of detecting a mutation [40]. ddPCR assays have a lower limit of detection and increased sensitivity compared to qPCR assay [41], allowing them to detect mutations at low concentrations that qPCR cannot.

Note that the false positive signals are different between ddPCR and qPCR. In qPCR, a false-positive amplification curve cannot be differentiated from a true-positive amplification curve. In contrast, a false-positive amplification droplet in ddPCR can be differentiated from true-positive amplification droplets (including both WT and MT) by locations of the clusters. Spurious positive droplets, if any, can be separated by properly choosing the cluster threshold (Figures S22 and S23). We did not observe non-specific signals in 60 cycles of ddPCR. This is in agreement with the literature [42,43], in which 55–60 cycles of ddPCR were used.

The presence of KRAS mutations in the ctDNA of pancreatic cancer patients has been shown to be indicative of progression free and overall survival [44]. Most studies focusing on a single or few time points frequently report either a low number of resectable PDAC patients expressing KRAS mutations in plasma [17,18] or substantially less than the number of positive tumor samples [45]. However, our results suggest that the majority of resectable PDAC patients will have a KRAS mutation in plasma at some time point during treatment, as we observed a KRAS mutation in five out of six patients that reached surgical resection. Three of these patients only had a detectable KRAS mutation present after baseline blood draw (Table 3). This result is not unexpected as increases in ctDNA over the course of treatment has been previously documented [34,44,46].

In one case (P004), a KRAS mutation was present in the plasma while we could not confirm it in the tumor. This patient had low cellularity within his/her tumor (Table 3), resulting in no mutations being detected. While this result is uncommon, the presence of ctDNA mutations in plasma without a corresponding tumor mutation has been demonstrated in the literature. Additionally, some evidence shows that the presence of ctDNA mutations may be more indicative of survival outcomes compared to tumor mutation status.

Previous studies on resectable PDAC yield different results on the relationship between the presence of ctDNA and clinical values. Singh et al. reported no relationship between KRAS mutation status and vascular encasement, tumor mass, or lymphatic invasion [17], but they did not stratify their analysis by resectable versus non-resectable patients. In contrast, a study of patients that qualified for pancreatectomy found that ctDNA was more frequently detected in higher AJCC T and N stages [20]. In our study, patients with T_1_ (P002 and P004) and T_3_ stages (P006 and P007) had a ctDNA mutation present at some time points. However, both T_1_ patients were detected with a KRAS mutation only at the start of treatment, while T_3_ patients were detected with a KRAS mutation multiple times past the baseline blood draw. It is possible that the appearance of ctDNA mutations over the course of treatment is indicative of tumor growth. We believe further investigation is warranted to determine if the presence of ctDNA at baseline or after baseline is a more important biomarker with regards to clinical values.

## 5. Conclusions

In summary, liquid biopsy is a promising tool in monitoring treatment response in various carcinomas, but it has not been well studied in resectable PDAC patients. Existing studies in the literature typically focus on a few individual time points such as baseline blood draw prior to treatment, post-treatment blood draw, or pre- and post-surgical blood draw. Our longitudinal study suggests that potentially valuable information in plasma KRAS mutations and sudden increases in cfDNA or CTC concentrations can happen throughout treatment. Spikes in cfDNA or changes in the KRAS mutation status of ctDNA may be predictive of the success of treatment in resectable PDAC patients undergoing neoadjuvant therapy and are associated with effective anti-cancer treatments resulting in cellular material dissemination. Further study is warranted to determine the significance of these measurements mid-treatment.

## Figures and Tables

**Table 1:** Summary of patient outcomes by the end of treatment (EoT).

Patient	Surgical Stage		Treatment Score	Radiation Therapy	EoT Outcome
	T	N			
P001	T_2_	N_1_	2	Yes	Completed treatment
P002	T_1c_	N_1_	2	No	Completed treatment
P003	N/A	N/A	2	No	Removed from treatment
P004	T_1b_	N_0_	1	Yes	Completed treatment
P005	T_2_	N_1_	3	No	Completed treatment
P006	T_3_	N_0_	2	Yes	Completed treatment
P007	T_3_	N_0_	1	Yes	Completed treatment

**Table 2:** CTCs, cfDNA concentration, and ctDNA prior to treatment.

Group	CTCs/mL	cfDNA (qPCR) ng/mL	cfDNA (ddPCR) ng/mL	ctDNA Positive
PDAC Survivors	<<0.5 (1/5)	6.3 ± 5.5	2.5 ± 1.2	0/7 qPCR 0/5 ddPCR
Cancer Patients	1.2 ± 1.2 (4/6)	7.8 ± 11.2	5.6 ± 2.4	0/7 qPCR 2/7 ddPCR

**Table 3:** KRAS mutation in tumor tissue or in plasma detected using ddPCR.

Patient ID	Tumor Mutation Cellularity (%MAF)	ddPCR (Cycle, %MAF)
P001	G12D 20% (6.8%)	Cycle 3, 0.33%
P002	G12D 40% (37.1%)	Baseline, 0.55%
P003	G12R 30% (8.2%)	
P004	Insufficient Tissue for Detection	Baseline, 1.04%
P005	G12V 40% (19.1%)	
P006	G12D 70% (52.1%)	Cycle 3, 0.19% Cycle 5, 0.66% Cycle 7, 0.21%
P007	G12D 20% (5.4%)	Cycle 3, 0.44% Cycle 8, 0.24%

## Data Availability

The data that support the findings of this study are available from the corresponding authors upon reasonable request.

## Supplementary Materials

The following are available online at: https://www.mdpi.com/article/10.3390/bios12040206/s1, Figure S1: The lateral filter array microfluidic (LFAM) device; Figure S2: Triple immunohistochemistry of a CTC. CTCs; Figure S3: Reference curve for wild-type KRAS; Figure S4: Mutation reference curves; Figure S5: Melt curve plots; Figure S6: CTC counts pooled; Figures S7–S13: CTC concentration over time for patient P001–P007; Table S1: List of adverse events in PDAC patients; Figures S14–S20: cfDNA concentration over time for patient P001–P007; Figure S21: KRAS mutation concordance between qPCR and ddPCR assays; Figure S22: ddPCR reaction scheme; and Figure S23: Examples of ddPCR results.

## References

[1] Luo J., Xiao L., Wu C., Zheng Y., Zhao N. (2013). The incidence and survival rate of population-based pancreatic cancer patients: Shanghai Cancer Registry 2004–2009. PLoS ONE.

[2] Versteijne E., Vogel J.A., Besselink M.G., Busch O.R., Wilmink J.W., Daams J.G., van Eijck C.H., Groot Koerkamp B., Rasch C.R., van Tienhoven G. (2018). Meta-analysis comparing upfront surgery with neoadjuvant treatment in patients with resectable or borderline resectable pancreatic cancer. Br. J. Surg..

[3] Heinrich S., Lang H. (2017). Neoadjuvant Therapy of Pancreatic Cancer: Definitions and Benefits. Int. J. Mol. Sci..

[4] Zeng S., Pöttler M., Lan B., Grützmann R., Pilarsky C., Yang H. (2019). Chemoresistance in Pancreatic Cancer. Int. J. Mol. Sci..

[5] Alix-Panabières C., Pantel K. (2021). Liquid Biopsy: From Discovery to Clinical Application. Cancer Discov..

[6] Bissolati M., Sandri M.T., Burtulo G., Zorzino L., Balzano G., Braga M. (2015). Portal vein-circulating tumor cells predict liver metastases in patients with resectable pancreatic cancer. Tumour Biol..

[7] Gemenetzis G., Groot V.P., Yu J., Ding D., Teinor J.A., Javed A.A., Wood L.D., Burkhart R.A., Cameron J.L., Makary M.A. (2018). Circulating Tumor Cells Dynamics in Pancreatic Adenocarcinoma Correlate With Disease Status: Results of the Prospective CLUSTER Study. Ann. Surg..

[8] Alix-Panabières C., Riethdorf S., Pantel K. (2008). Circulating tumor cells and bone marrow micrometastasis. Clin. Cancer Res..

[9] Van der Meer P.F., de Wildt-Eggen J. (2006). The effect of whole-blood storage time on the number of white cells and platelets in whole blood and in white cell-reduced red cells. Transfusion.

[10] Chen K., Amontree J., Varillas J., Zhang J., George T.J., Fan Z.H. (2020). Incorporation of lateral microfiltration with immunoaffinity for enhancing the capture efficiency of rare cells. Sci. Rep..

[11] Chen K., Dopico P., Varillas J., Zhang J., George T.J., Fan Z.H. (2019). Integration of Lateral Filter Arrays with Immunoaffinity for Circulating-Tumor-Cell Isolation. Angew. Chem. Int. Ed. Engl..

[12] Allard W.J., Matera J., Miller M.C., Repollet M., Connelly M.C., Rao C., Tibbe A.G., Uhr J.W., Terstappen L.W. (2004). Tumor cells circulate in the peripheral blood of all major carcinomas but not in healthy subjects or patients with nonmalignant diseases. Clin. Cancer Res..

[13] Sandri M.T., Zorzino L., Cassatella M.C., Bassi F., Luini A., Casadio C., Botteri E., Rotmensz N., Adamoli L., Nolè F. (2010). Changes in circulating tumor cell detection in patients with localized breast cancer before and after surgery. Ann. Surg. Oncol..

[14] Li P., Stratton Z.S., Dao M., Ritz J., Huang T.J. (2013). Probing circulating tumor cells in microfluidics. Lab Chip.

[15] Wang L., Balasubramanian P., Chen A.P., Kummar S., Evrard Y.A., Kinders R.J. (2016). Promise and limits of the CellSearch platform for evaluating pharmacodynamics in circulating tumor cells. Semin. Oncol..

[16] Cheng C., Omura-Minamisawa M., Kang Y., Hara T., Koike I., Inoue T. (2009). Quantification of circulating cell-free DNA in the plasma of cancer patients during radiation therapy. Cancer Sci..

[17] Singh N., Gupta S., Pandey R.M., Chauhan S.S., Saraya A. (2015). High levels of cell-free circulating nucleic acids in pancreatic cancer are associated with vascular encasement, metastasis and poor survival. Cancer Investig..

[18] Pietrasz D., Pécuchet N., Garlan F., Didelot A., Dubreuil O., Doat S., Imbert-Bismut F., Karoui M., Vaillant J.C., Taly V. (2017). Plasma Circulating Tumor DNA in Pancreatic Cancer Patients Is a Prognostic Marker. Clin. Cancer Res..

[19] Earl J., Garcia-Nieto S., Martinez-Avila J.C., Montans J., Sanjuanbenito A., Rodríguez-Garrote M., Lisa E., Mendía E., Lobo E., Malats N. (2015). Circulating tumor cells (Ctc) and kras mutant circulating free Dna (cfdna) detection in peripheral blood as biomarkers in patients diagnosed with exocrine pancreatic cancer. BMC Cancer.

[20] Groot V.P., Mosier S., Javed A.A., Teinor J.A., Gemenetzis G., Ding D., Haley L.M., Yu J., Burkhart R.A., Hasanain A. (2019). Circulating Tumor DNA as a Clinical Test in Resected Pancreatic Cancer. Clin. Cancer Res..

[21] Varillas J.I., Zhang J., Chen K., Barnes I.I., Liu C., George T.J., Fan Z.H. (2019). Microfluidic Isolation of Circulating Tumor Cells and Cancer Stem-Like Cells from Patients with Pancreatic Ductal Adenocarcinoma. Theranostics.

[22] Van Roessel S., Janssen B.V., Soer E.C., Fariña Sarasqueta A., Verbeke C.S., Luchini C., Brosens L.A.A., Verheij J., Besselink M.G. (2021). Scoring of tumour response after neoadjuvant therapy in resected pancreatic cancer: Systematic review. Br. J. Surg..

[23] Sanjay K., Shi C., Adsay V., Fitzgibbons P., Frankel W., Klimstra D., Kraninskas A., Mino-Kenudson M., Pawlik T., Vauthey J.-N. (2017). Protocol for the Examination of Specimens from Patients with Carcinoma of the Exocrine Pancreas.

[24] (2017). Common Terminology Criteria for Adverse Events (CTCAE) Version 5.0.

[25] Deer E.L., González-Hernández J., Coursen J.D., Shea J.E., Ngatia J., Scaife C.L., Firpo M.A., Mulvihill S.J. (2010). Phenotype and genotype of pancreatic cancer cell lines. Pancreas.

[26] Eckert L.B., Repasky G.A., Ulkü A.S., McFall A., Zhou H., Sartor C.I., Der C.J. (2004). Involvement of Ras activation in human breast cancer cell signaling, invasion, and anoikis. Cancer Res..

[27] Cicenas J., Kvederaviciute K., Meskinyte I., Meskinyte-Kausiliene E., Skeberdyte A. (2017). KRAS, TP53, CDKN2A, SMAD4, BRCA1, and BRCA2 Mutations in Pancreatic Cancer. Cancers.

[28] Thierry A.R., Mouliere F., El Messaoudi S., Mollevi C., Lopez-Crapez E., Rolet F., Gillet B., Gongora C., Dechelotte P., Robert B. (2014). Clinical validation of the detection of KRAS and BRAF mutations from circulating tumor DNA. Nat. Med..

[29] Bustin S.A., Benes V., Garson J.A., Hellemans J., Huggett J., Kubista M., Mueller R., Nolan T., Pfaffl M.W., Shipley G.L. (2009). The MIQE guidelines: Minimum information for publication of quantitative real-time PCR experiments. Clin. Chem..

[30] Hugenschmidt H., Labori K.J., Brunborg C., Verbeke C.S., Seeberg L.T., Schirmer C.B., Renolen A., Borgen E.F., Naume B., Wiedswang G. (2020). Circulating Tumor Cells are an Independent Predictor of Shorter Survival in Patients Undergoing Resection for Pancreatic and Periampullary Adenocarcinoma. Ann. Surg..

[31] Court C.M., Ankeny J.S., Sho S., Winograd P., Hou S., Song M., Wainberg Z.A., Girgis M.D., Graeber T.G., Agopian V.G. (2018). Circulating Tumor Cells Predict Occult Metastatic Disease and Prognosis in Pancreatic Cancer. Ann. Surg. Oncol..

[32] Lu Y.T., Zhao L., Shen Q., Garcia M.A., Wu D., Hou S., Song M., Xu X., Ouyang W.H., Ouyang W.W. (2013). NanoVelcro Chip for CTC enumeration in prostate cancer patients. Methods.

[33] Gold M., Pachmann K., Kiani A., Schobert R. (2021). Monitoring of circulating epithelial tumor cells using the Maintrac. Mol. Clin. Oncol..

[34] Gerratana L., Davis A.A., Zhang Q., Basile D., Rossi G., Strickland K., Franzoni A., Allegri L., Mu Z., Zhang Y. (2021). Longitudinal Dynamics of Circulating Tumor Cells and Circulating Tumor DNA for Treatment Monitoring in Metastatic Breast Cancer. JCO Precis. Oncol..

[35] Wang Y., Kim T.H., Fouladdel S., Zhang Z., Soni P., Qin A., Zhao L., Azizi E., Lawrence T.S., Ramnath N. (2019). PD-L1 Expression in Circulating Tumor Cells Increases during Radio(chemo)therapy and Indicates Poor Prognosis in Non-small Cell Lung Cancer. Sci. Rep..

[36] Arigami T., Uenosono Y., Yanagita S., Okubo K., Kijima T., Matsushita D., Amatatsu M., Kurahara H., Maemura K., Natsugoe S. (2017). Clinical significance of circulating tumor cells in blood from patients with gastric cancer. Ann. Gastroenterol. Surg..

[37] Okubo K., Uenosono Y., Arigami T., Mataki Y., Matsushita D., Yanagita S., Kurahara H., Sakoda M., Kijima Y., Maemura K. (2017). Clinical impact of circulating tumor cells and therapy response in pancreatic cancer. Eur. J. Surg. Oncol..

[38] Dorsey J.F., Kao G.D., MacArthur K.M., Ju M., Steinmetz D., Wileyto E.P., Simone C.B., Hahn S.M. (2015). Tracking viable circulating tumor cells (CTCs) in the peripheral blood of non-small cell lung cancer (NSCLC) patients undergoing definitive radiation therapy: Pilot study results. Cancer.

[39] Swystun L.L., Mukherjee S., Liaw P.C. (2011). Breast cancer chemotherapy induces the release of cell-free DNA, a novel procoagulant stimulus. J. Thromb. Haemost..

[40] Zhong Y., Zhou Q., Zhang Y., Zhou S., Zhang G., Jiang C., Zhang Z., Zhang X., Xu J., Jin C. (2020). Cell-free DNA as a biomarker for colorectal cancer: A retrospective analysis in patients before and after surgery. Cell. Mol. Biol..

[41] Taylor S.C., Laperriere G., Germain H. (2017). Droplet Digital PCR versus qPCR for gene expression analysis with low abundant targets: From variable nonsense to publication quality data. Sci. Rep..

[42] Hindson B.J., Ness K.D., Masquelier D.A., Belgrader P., Heredia N.J., Makarewicz A.J., Bright I.J., Lucero M.Y., Hiddessen A.L., Legler T.C. (2011). High-Throughput Droplet Digital PCR System for Absolute Quantitation of DNA Copy Number. Anal. Chem..

[43] Witte A.K., Mester P., Fister S., Witte M., Schoder D., Rossmanith P. (2016). A Systematic In-vestigation of Parameters Influencing Droplet Rain in the Listeria monocytogenes prfA Assay-Reduction of Ambiguous Results in ddPCR. PLoS ONE.

[44] Bernard V., Kim D.U., San Lucas F.A., Castillo J., Allenson K., Mulu F.C., Stephens B.M., Huang J., Semaan A., Guerrero P.A. (2019). Circulating Nucleic Acids Are Associated With Outcomes of Patients With Pancreatic Cancer. Gastroenterology.

[45] Patel H., Okamura R., Fanta P., Patel C., Lanman R.B., Raymond V.M., Kato S., Kurzrock R. (2019). Clinical correlates of blood-derived circulating tumor DNA in pancreatic cancer. J. Hematol. Oncol..

[46] Choudhury A.D., Werner L., Francini E., Wei X.X., Ha G., Freeman S.S., Rhoades J., Reed S.C., Gydush G., Rotem D. (2018). Tumor fraction in cell-free DNA as a biomarker in prostate cancer. JCI Insight.

