# Supplementary material for: Longitudinal Study of Circulating Biomarkers in Patients with Resectable Pancreatic Ductal Adenocarcinoma
Source: Biosensors (Basel). 2022 Mar 30;12(4):206. doi: 10.3390/bios12040206 (PMC9028387; doi:10.3390/bios12040206)
Supplement: Supplementary file 1 [file biosensors-12-00206-s001.zip › biosensors-1604585-supplementary.pdf]

biosensors

MDPI

Article

Longitudinal Study of Circulating Biomarkers in Patients with Resectable Pancreatic Ductal Adenocarcinoma

Pablo J. Dopico [1], Minh-Chau N. Le [1], Benjamin Burgess [2], Zhijie Yang [3], Yu Zhao [3], Youxiang Wang [3], Thomas J. George [2,4,*] and Z. Hugh Fan [1,2,5,*]

1 Interdisciplinary Microsystems Group, Department of Mechanical and Aerospace Engineering, University of Florida, P.O. Box 116250, Gainesville, FL 32611, USA; ufpablo@ufl.edu (P.J.D.); minhchaunle@ufl.edu (M.-C.N.L.);
2 UF Health Cancer Center, University of Florida, 2033 Mowry Rd., Gainesville, FL 32610, USA; benjamin.burgess@ufl.edu
3 Atila Biosystems, 740 Sierra Vista Ave., Unit E, Mountain View, CA 94043, USA; zhijie.yang@atilabiosystems.com (Z.Y.); shoemakerzhao@gmail.com (Y.Z.); ywang@atilabiosystems.com (Y.W.)
4 Department of Medicine, University of Florida, 1600 SW Archer Rd., Gainesville, FL 32610, USA
5 J. Crayton Pruitt Family Department of Biomedical Engineering, University of Florida, Gainesville, FL 32611, USA
* Correspondence: thom.george@medicine.ufl.edu (T.J.G.); hfan@ufl.edu (Z.H.F.)

Citation: Dopico, P.J.; Le, M.-C.N.; Burgess, B.; Yang, Z.; Zhao, Y.; Wang, Y.; George, T.J.; Fan, H.Z. Longitudinal Study of Circulating Biomarkers in Patients with Resectable Pancreatic Ductal Adenocarcinoma. *Biosensors* **2022**, *12*, 206. https://doi.org/10.3390/bios12040206

Received: 3 February 2022
Accepted: 28 March 2022
Published: 30 March 2022

Publisher's Note: MDPI stays neutral with regard to jurisdictional claims in published maps and institutional affiliations.

Copyright: © 2022 by the authors. Licensee MDPI, Basel, Switzerland. This article is an open access article distributed under the terms and conditions of the Creative Commons Attribution (CC BY) license (https://creativecommons.org/licenses/by/4.0/).

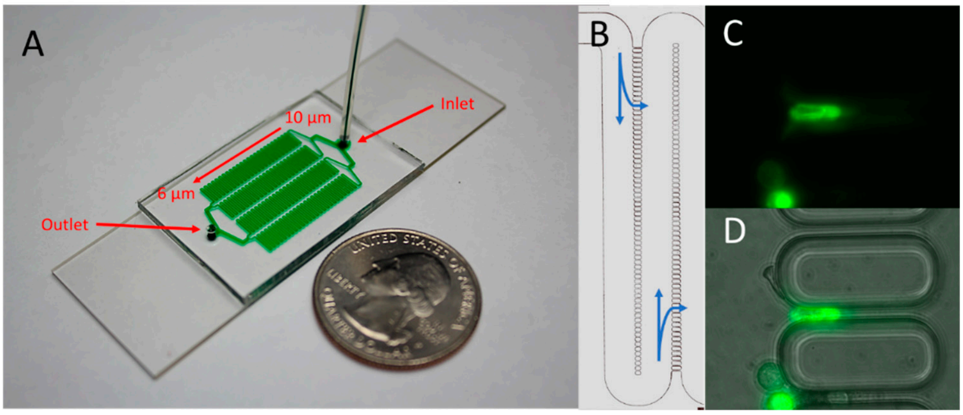

Figure S1. The lateral filter array microfluidic (LFAM) device (A), consists of serpentine channels made of lateral filters (B). The filter size decreases from 10 µm near the inlet to 6 µm near the outlet. The arrows in (B) indicate the direction of a main flow along the serpentine channel and a lateral flow along the filters. CaPan-1 cells stained with Vybrant DiD (Invitrogen, Carlsbad, CA) are captured either inside a filter (via both filtration and immunoaffinity mechanisms) or outside a filter (via immunoaffinity only) and imaged with fluorescence (C) and brightfield (D).

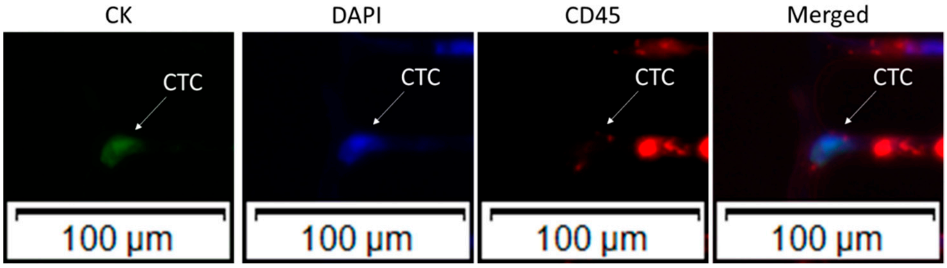

Figure S2. Triple immunohistochemistry of a CTC. CTCs were defined as CK^+ (Green), $DAPI^+$ (blue), and $CD45^-$ (Red). A scale bar of 100 µm is provide with each image.

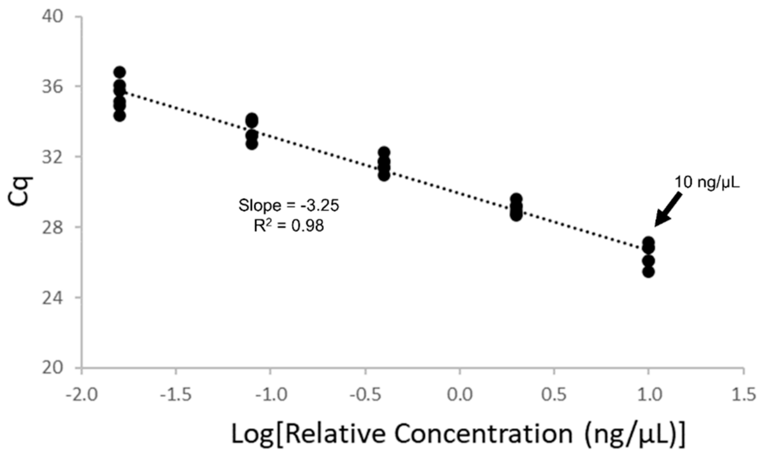

Figure S3. Reference curve for wild-type KRAS. Six wells were run for each concentration and represented by individual points in the plot.

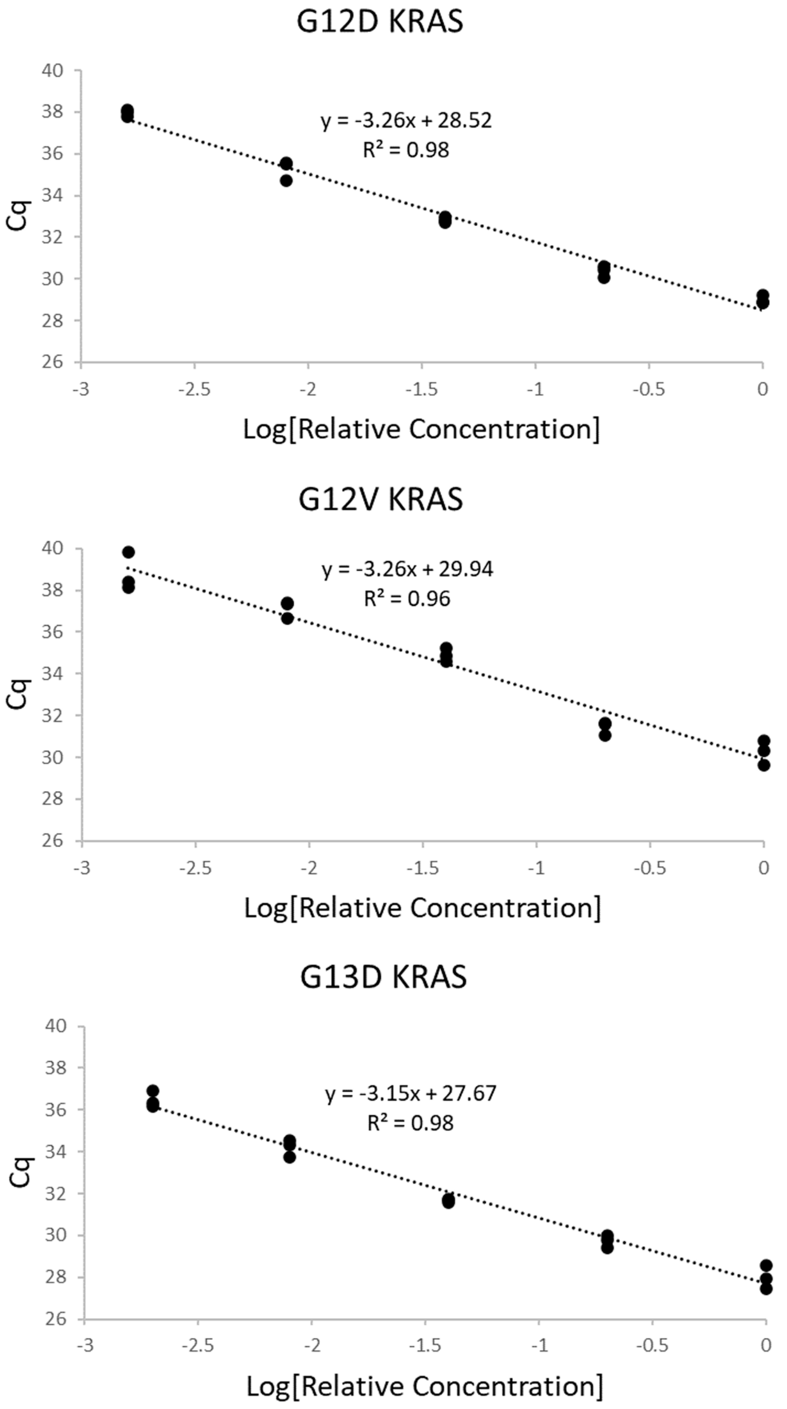

Figure S4. Mutation reference curves using DNA from a G12D standard reference (top), CaPan-1 cells for G12V (middle), or MDA-MB-231 cells for G13D (bottom).

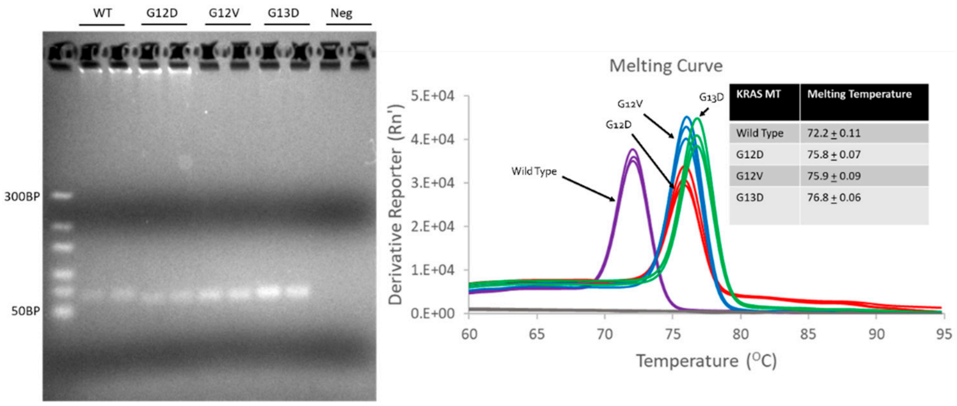

Figure S5. Melt curve plots (right) of wild-type (purple), G12D (red), G12V (blue), and G13D (green) of positive controls amplified by qPCR. Positive control melt curve experiments were repeated three times and the melting temperatures between experiments were averaged (in the table inset), indicating extremely small inter-experiment variability. The PCR product size was confirmed utilizing gel electrophoresis (left).

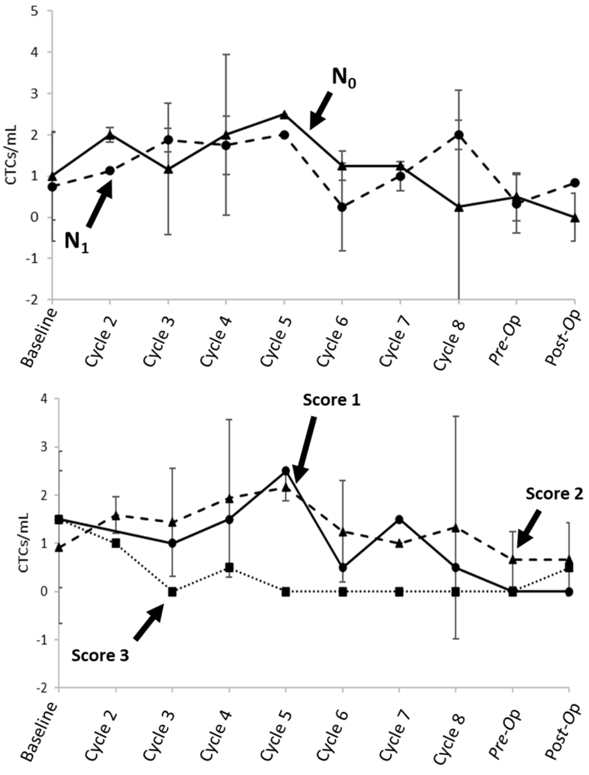

Figure S6. CTC counts pooled by the presence of lymph node invasion (top) at surgical staging or treatment score (bottom).

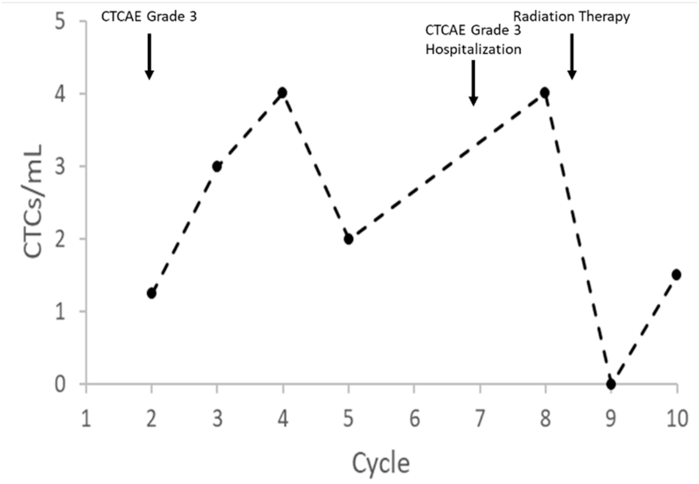

Figure S7. CTC concentration over time for patient P001. Common terminology for adverse events (CTCAE) grade 3 or higher were indicated in the figure.

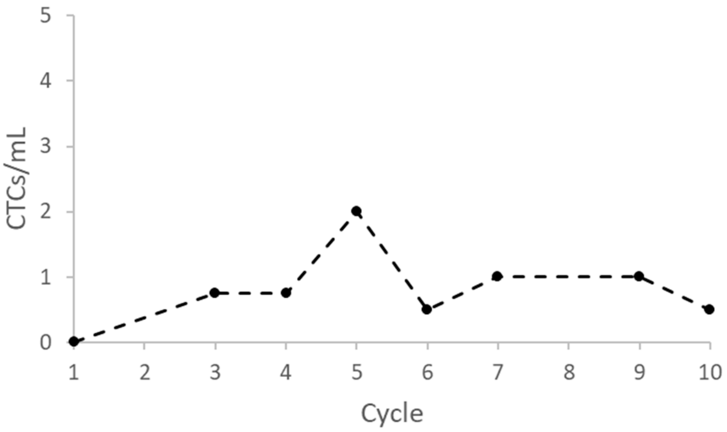

Figure S8. CTC concentration over time for P002.

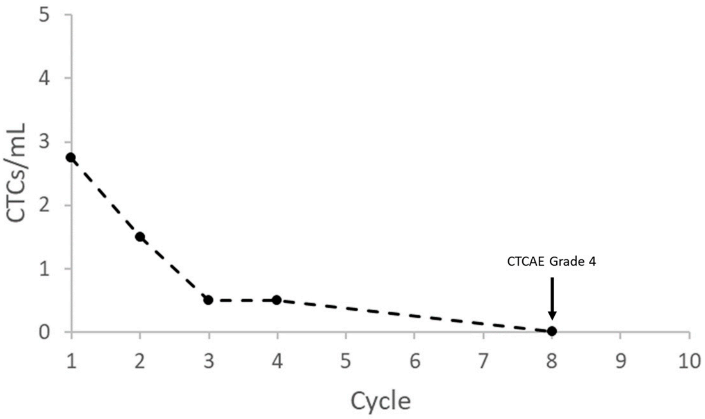

Figure S9. CTC concentration over time for P003.

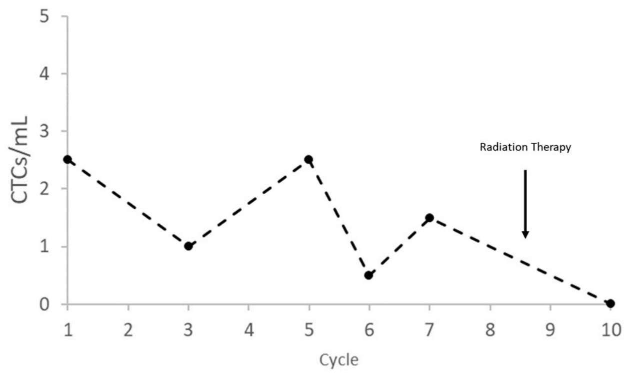

Figure S10. CTC concentration over time for P004.

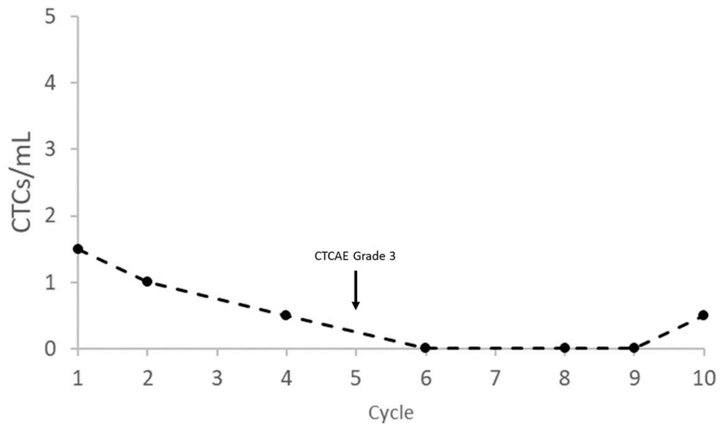

Figure S11. CTC concentration over time for P005.

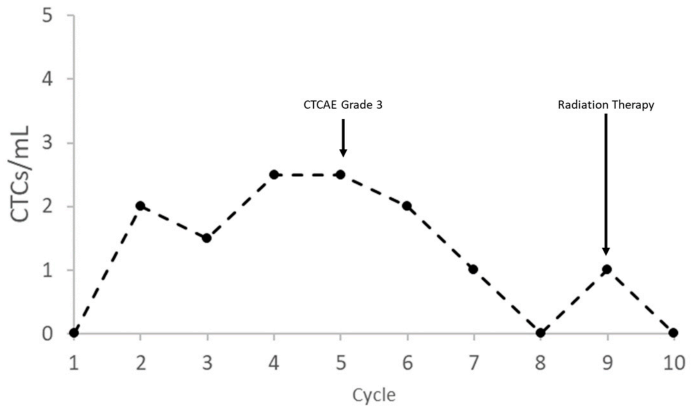

Figure S12. CTC concentration over time for P006.

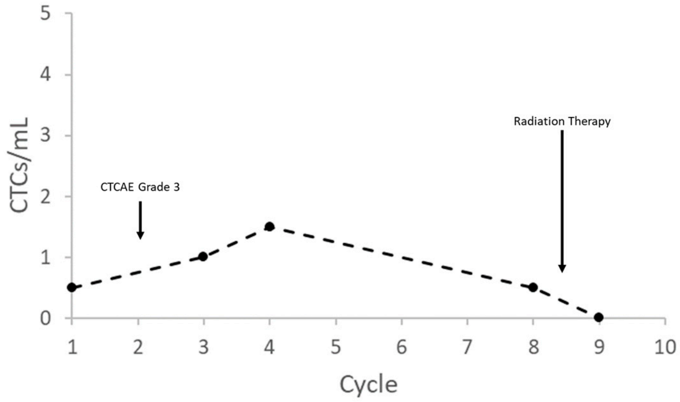

Figure S13. CTC concentration over time for P007.

Table S1. List of CTCAE of grade 3 or higher in PDAC patients at various treatment cycles.

Patient	List of CTCAE Events (Cycle – Grade – Description)
P001	C_2 – 3 – Diarrhea<br>C_7 – 3 – Pancreatitis, Hypokalemia, Aspartate and Alanine aminotransferase increased
P002	None
P003	C_4 – 3 – Fatigue, weight loss, neutrophil count decreased<br>EoT – 3 – Alkaline phosphatase increased, anorexia, dehydration, diarrhea, electrocardiogram QT corrected interval prolonged, hypotension<br>EoT – 4 – Colitis, hypokalemia, lower gastrointestinal hemmorhage
P004	None
P005	C_5 – 3 – Other: laboratory abnormality
P006	C_5 – 3 – Neutrophil count decreased
P007	C_2 – 3 – Abdominal pain, vomiting, hyponatremia, anorexia

CTCAE: common terminology criteria for adverse events. PDAC: pancreatic ductal adenocarcinoma. C: the number of treatment cycles. EOT: end of treatment.

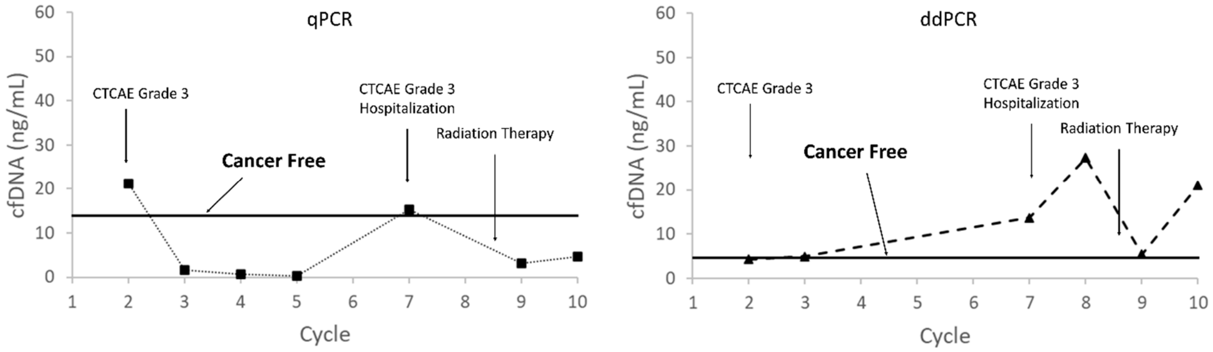

Figure S14. cfDNA concentration over time for patient P001 according to qPCR (left) and ddPCR (right). The solid, horizontal line indicates the maximum cfDNA concentration detected in the PDAC survivor controls. Cytotoxic events with a CTCEA score of three or greater were indicated.

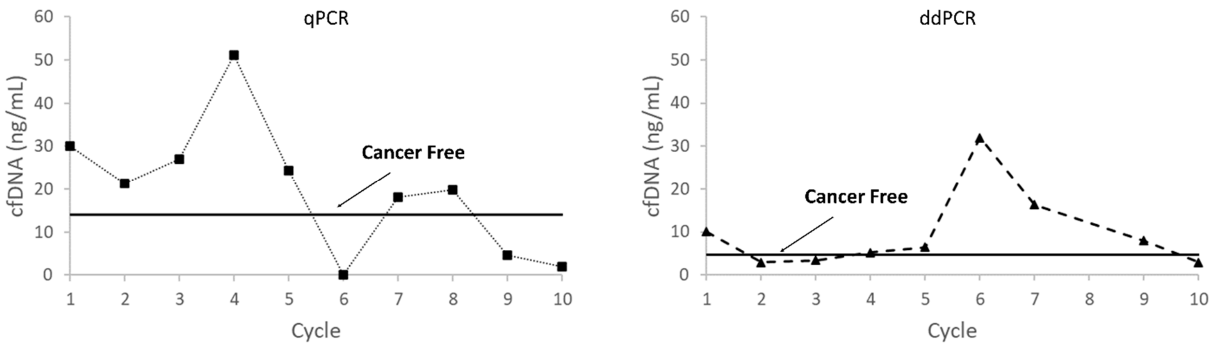

Figure S15. cfDNA concentration over time for P002 according to qPCR (left) and ddPCR (right).

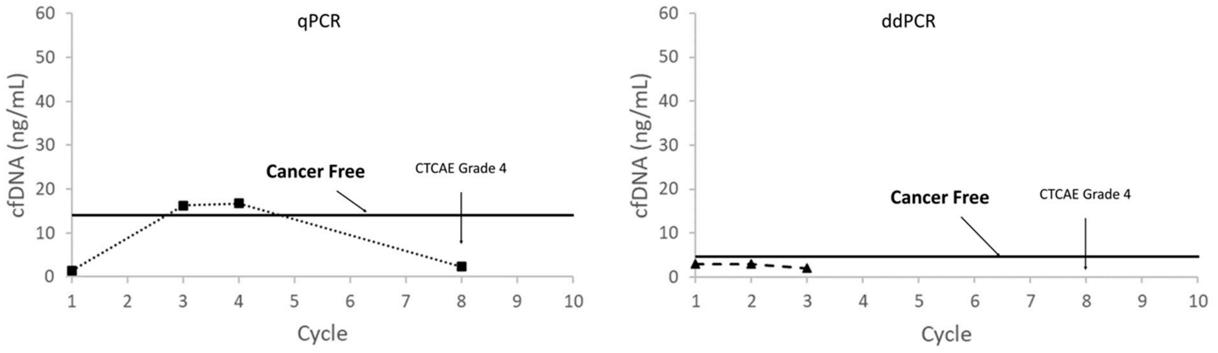

Figure S16. cfDNA concentration over time for P003 according to qPCR (left) and ddPCR (right).

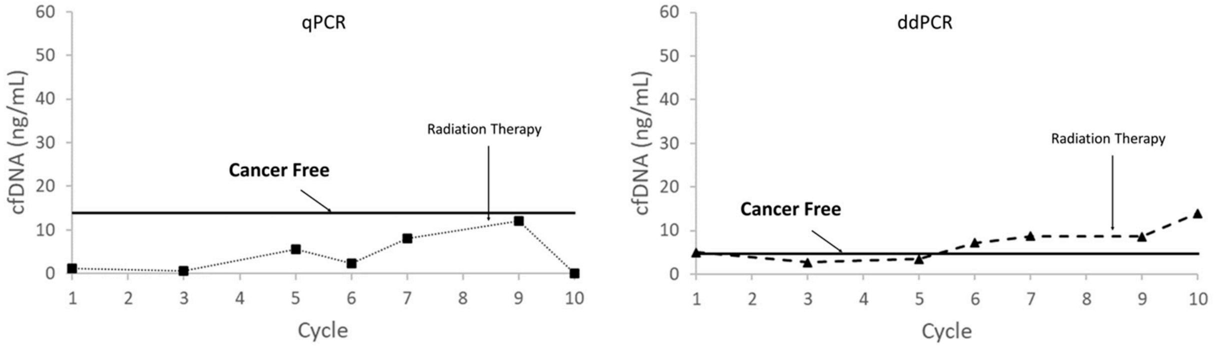

Figure S17. cfDNA concentration over time for P004 according to qPCR (left) and ddPCR (right).

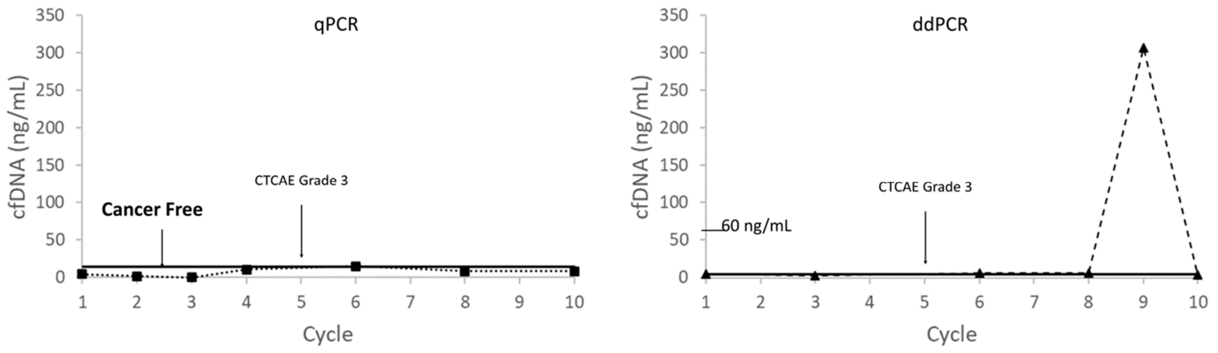

Figure S18. cfDNA concentration over time for P005 according to qPCR (left) and ddPCR (right).

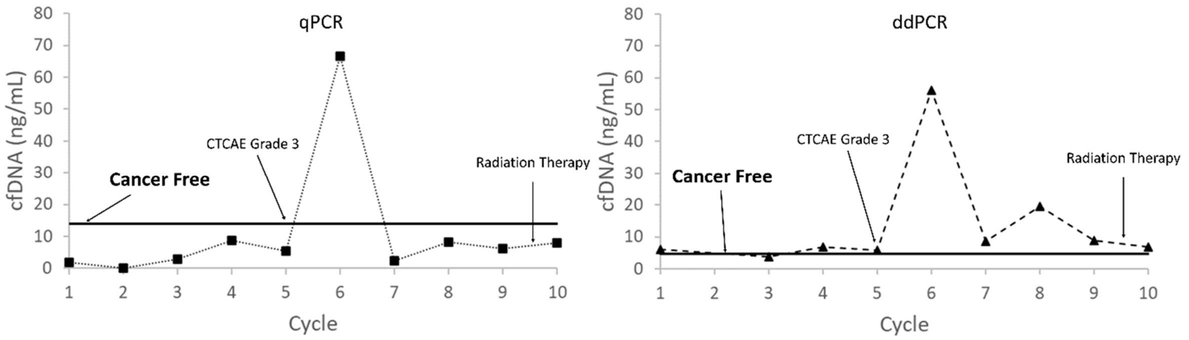

Figure S19. cfDNA concentration over time for P006 according to qPCR (left) and ddPCR (right).

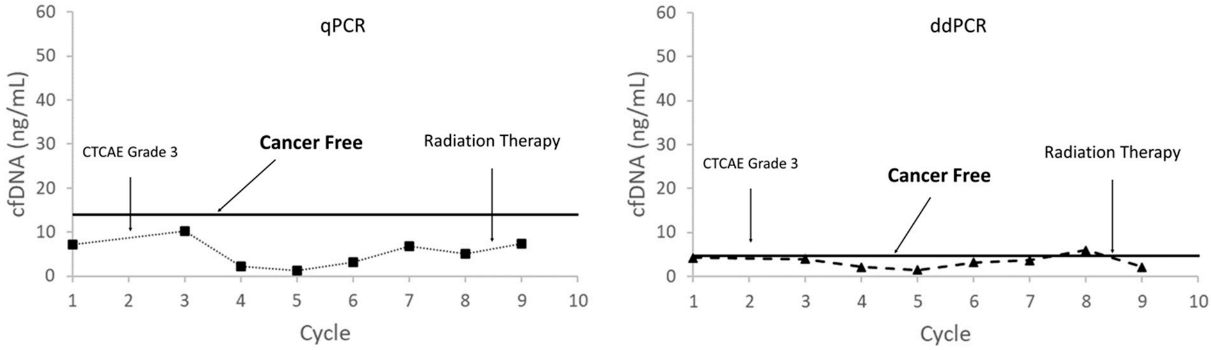

Figure S20. cfDNA concentration over time for P007 according to qPCR (left) and ddPCR (right).

Patient	Tumor MT	qPCR MT	ddPCR MT
CTC001	-	-	-
CTC002	-	-	-
CTC003	G12V	G12V	+
CTC004	-	-	-
CTC005	-	-	-
CTC006	-	-	-
CTC014	KRAS	-	-
CTC022	G12S	-	-
CTC027	G12V	-	-
CTC028	KRAS	-	-
CTC030	-	-	-
CTC032	-	G13D	+
CTC039	-	-	-
CTC041	-	-	-
CTC043	-	-	-
CTC051	KRAS	-	-
CTC052	KRAS	G12D	+
CTC053	-	-	-

Figure S21. KRAS mutation concordance between qPCR and ddPCR assays using plasma samples from CRC patients. "+" indicates mutation was detected while "-" was not.

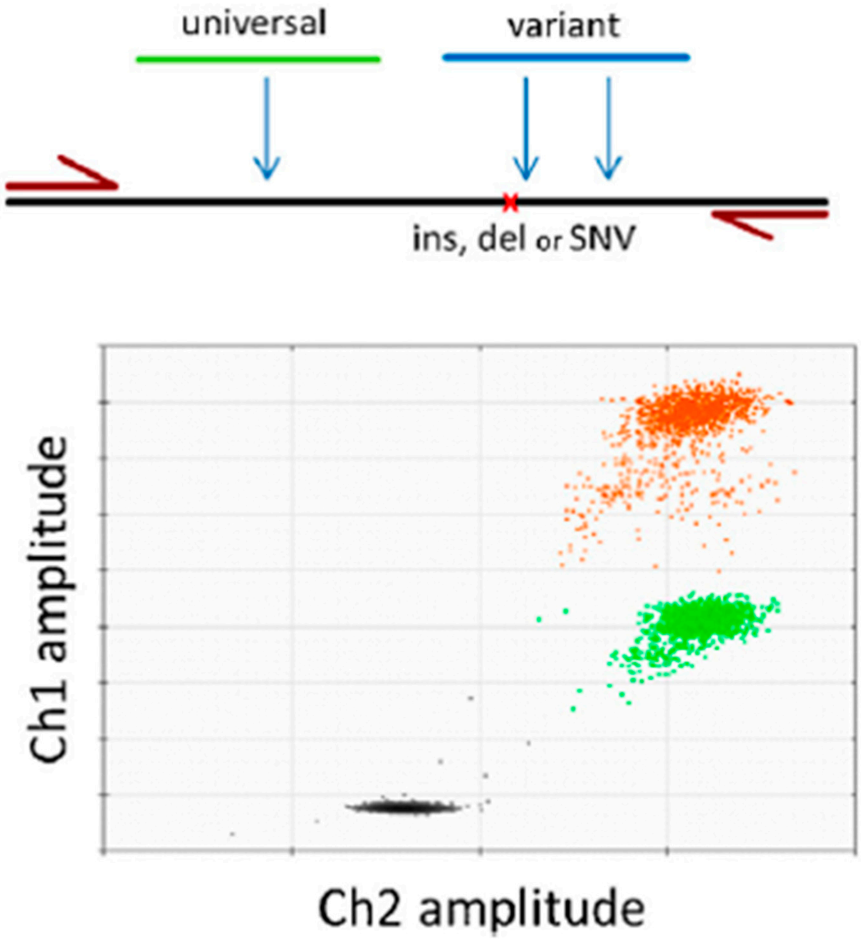

Figure S22. (top) Duplex reaction scheme used for ddPCR, illustrating the primer and probe hybridization arrangement. The universal probe (or wild type in the main text of this manuscript) targets an area of the amplicon that is not expected to be variable and thus, provides a reference for the total number of molecules present in the sample irrespective of sequence. The variant probe (or mutant type in the main text of this manuscript) targets the area of the amplicon that is expected to contain one or more variants, including insertions (ins), deletions (del), and single nucleotide variants (SNV). (bottom) Example of the configuration of clusters in the 2D plot. The amplitude in channel 1 (ch1) is represented on the y-axis with the amplitude in channel 2 (ch2) represented on the x-axis. Three clusters are identified as single-positive for Ch2 (green), double-positive (orange), and double-negative (grey). This figure is adapted from the reference, Whale et al., *Biomol. Detect. Quantif.* 2016, 10, 15–23.

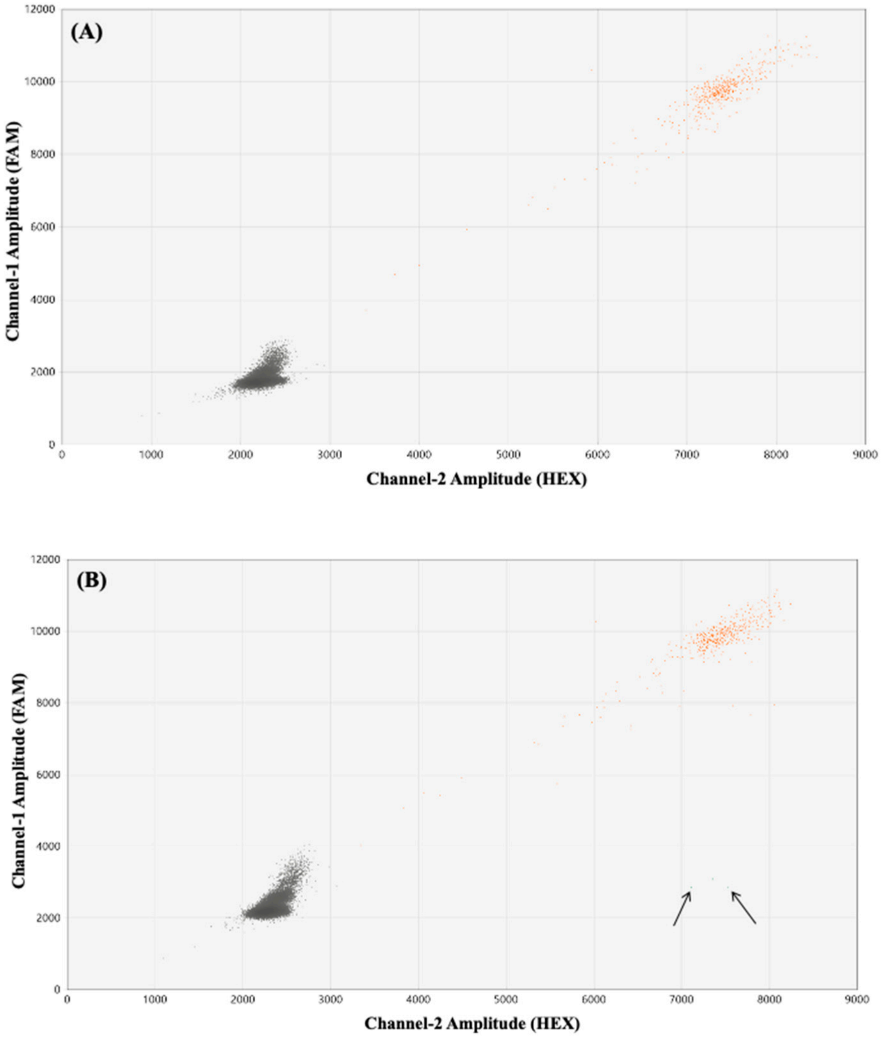

Figure S23. Two examples of ddPCR result from BioRad QuantaSoft Analysis Pro, showing the fluorescence signals of the HEX channel and the FAM channel (A: P001, C7, no mutation; B: P006, C5, mutation detected). Each dot is a droplet. Gray dots on the bottom left are negative droplets. Orange dots on the top right are wild-type droplets. Green dots on the bottom right, indicated by arrows, are mutation droplets.
